# Assessing Spatial Accessibility to Primary Health Care Services in Beijing, China

**DOI:** 10.3390/ijerph182413182

**Published:** 2021-12-14

**Authors:** Jiawei Zhang, Peien Han, Yan Sun, Jingyu Zhao, Li Yang

**Affiliations:** Department of Health Policy and Management, School of Public Health, Peking University, Beijing 100191, China; jiaweizhang1997@126.com (J.Z.); 1610306118@pku.edu.cn (P.H.); sunyanedu@126.com (Y.S.); 13683096077@163.com (J.Z.)

**Keywords:** spatial accessibility, modified two-step floating catchment area (M2SFCA) method, primary health care institutions, Beijing

## Abstract

Primary health care has been emphasized as a pillar of China’s current round of health reforms throughout the previous decade. The purpose of this study is to analyze the accessibility of primary health care services in Beijing and to identify locations with a relative scarcity of health personnel. Seven ecological conservation districts, which are relatively underdeveloped, were selected in the study. The Gini coefficient and Lorenz curve, as well as the shortest trip time and modified two-step floating catchment area (M2SFCA) approach, are used to quantify inequalities in primary health care resources and spatial accessibility. The Gini coefficient of primary medical services was calculated as high as 0.705, showing a significant disparity in primary care services. A total of 81.22% of communities reached the nearest primary care institution within 15 min. The average accessibility of primary healthcare services, as measured by the number of health professionals per 1000 population, was 2.34 in the 1715 communities of seven ecological conservation districts. Three hundred and ninety-one communities (22.80%) were identified with relatively low accessibility. More primary health professionals should be allocated to Miyun, Mentougou, and Changping Districts. Overall, the primary healthcare resources were distributed unevenly in most districts. According to our study, expanding primary healthcare institutions, increasing the number of competent health professionals, and enhancing road networks will all be effective ways to increase spatial accessibility and reduce primary healthcare service disparity in Beijing.

## 1. Introduction

**Figure 1:**
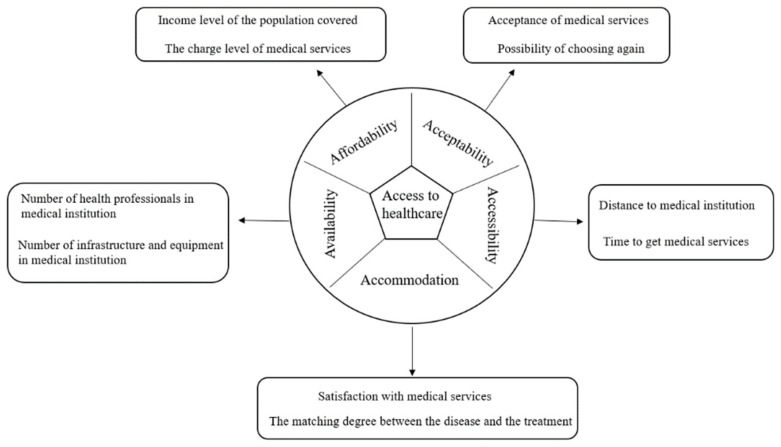
5A concept of access to healthcare.

In 1981, Penchansky and Thomas defined [1] the influencing factors of medical services as “5 A”s, namely availability, accessibility, affordability, acceptability, and accommodation Figure 1. Accessibility mainly considers spatial accessibility and nonspatial accessibility [2]. Spatial accessibility is primarily concerned with the distribution of population (demand) and medical facilities (supply) in space. Nonspatial accessibility takes into account demographic and socioeconomic variables such as age, gender, and income. As demand for medical services grows and medical services acquire more distinct spatial characteristics, an increasing number of studies have examined the equity and rationality of medical resource allocation, with the research findings providing more realistic guidance for policymakers [3]. The spatial distribution of medical services has a significant impact on residents’ access to medical services.

To be more specific, accessibility assesses the potential for receiving health services and can be determined through a comprehensive analysis of facilities, the population’s geographic distribution, and transportation [4]. In recent years, an increasing number of studies have focused on measuring spatial accessibility, in which Geographic Information System (GIS) technology has been widely used [5]. GIS can easily collect, store, and analyze spatial location data, which has advantages in calculating the geographic accessibility of medical services, putting forward more scientific suggestions for the development and construction of medical services.

In the Declaration of Alma-Ata, primary health care is emphasized as “the first level of contact of individuals, the family, and community with the national health system bringing health care as close as possible to where people live and work, and constitutes the first element of a continuing health care process [6].” With the rapid development of China’s economy, the new direction of national development has shifted to meeting residents’ expectations for a better life, of which healthcare services are essential components.

The Chinese government launched a new round of reforms to expand its healthcare and health system in 2009 and establishing a primary health-care-based health integrated delivery system is highlighted in the reform [7]. The government has increased input to subsidize declining medical revenues in primary health care institutions, and thus promote access to health care [8]. However, although primary health care institutions constitute more than 94% of all health institutions, 60.2% of health professionals are employed in hospitals and most of the health resources are allocated to secondary and tertiary hospitals [9]. In addition, hospitals are developing more rapidly than primary health care institutions, which could further enlarge the capacity gap and jeopardize governmental achievements in strengthening primary health care [10]. Patients tend to seek services at secondary and tertiary hospitals, despite longer travel time, and primary health services are in a vulnerable position, which is ineffective [11]. To tackle this problem, the Chinese government proposed a hierarchical system to enhance primary health care services’ capacity to keep the system operating effectively. Through the “first visit” principle and “two-way” referral system in primary care facilities, medical consultation behavior is better organized, ultimately maximizing the use of medical resources. Improving the accessibility and capacity of primary health care facilities is the best way to build a hierarchical system of diagnosis and treatment.

At the same time, the Opinions on Promoting the Gradual Equalization of Basic Public Health Services proposed the concept of “equalization of basic public health services” and called for promoting the access of urban and rural residents to equalized essential public health services to meet their basic health needs. China issued the “Healthy China 2030 Planning Outline” in October 2016, which revealed concerns about the equalization of primary health services at the national level [12]. 

As the capital with many national medical centers, Beijing has rapidly grown into a megacity with more than 20 million people. The health system in Beijing has been with complete function and is among the first ranks in the world. It has 11,211 medical institutions, 137,239 beds and 375,673 health professionals in 2020. However, there are still problems such as the shortage of health care services, especially primary health care services, and the structural imbalance of health resources [13]. As a result, the government faces significant challenges in providing adequate health care services to such a large population. To improve the capacity of primary healthcare institutions and achieve the goal of equalization of primary health services, the government of Beijing released the “Health Beijing 2030 Planning Outline”, which proposes to improve the primary health care service network and create a 15-min primary health care service zone in September 2017. Based on that, the Beijing Municipal Commission of Health and Family Planning formulated the “2019 Beijing Basic Health Essentials” to optimize the community setting plan and improve the 15-min community health service zone according to the status of the resident population and the construction of primary health institutions in 2019. A precise assessment of the current distribution of healthcare resources is critical for identifying and addressing existing inequities in primary health care services, ultimately minimizing disparities in primary health care services. Typically, while evaluating a region’s medical service capacity, three elements must be considered: the supply of medical services, the residents’ medical requirements, and the cost of residents receiving medical treatment. All three aspects are considered ‘accessibility’ in this article. This accessibility is broader than the 5A model’s idea of “accessibility,” and we might refer to it as ‘generalized accessibility.’ This generalized accessibility is related to the preceding 5A model’s availability and accessibility. Availability refers to the provision of medical services, which is quantified in this study by the number of health professionals in each primary medical institution. Accessibility is measured in terms of the distance to the medical facility and the amount of time required to complete the travel. The former is expressed in terms of the time required to reach the nearest medical facility, whereas the latter is expressed in terms of the journey distance divided by the travel speed used in the generalized accessibility calculation Finally, we evaluate the aggregate demand of inhabitants and reflect it in this text through the population of each residential community. This study estimated the generalized spatial accessibility for which we are concerned by combining the aforementioned variables.

Most previous studies on spatial accessibility in China have been conducted at the street or township level [14,15,16,17]. This type of large-scale study, however, is insufficient to give effective data to assist policymakers in developing specific hospital construction plans. Analyzing spatial accessibility at a finer scale, for example, at the community level, might result in more precise estimates and may aid in the planning of health care services.

This study used the modified two-step floating catchment area (M2SFCA) method to assess the spatial accessibility of seven districts in Beijing and set the time threshold at 15 min to identify the relative health workforce shortage areas, which may assist the government or planners in monitoring spatial inequalities in primary health care services and informing strategies for further improvements to medical services.

## 2. Materials and Methods

### 2.1. Study Area

**Figure 2:**
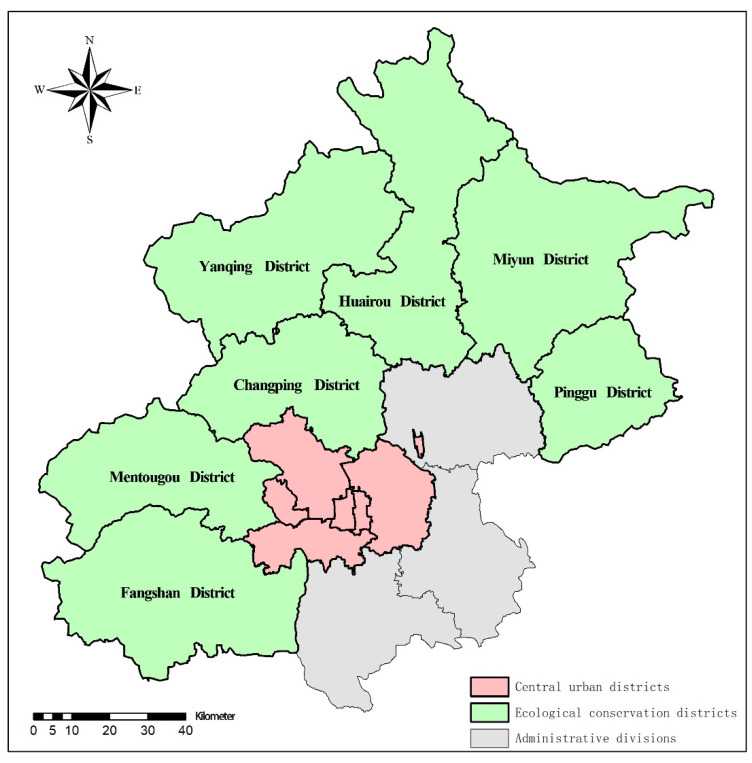
Functional orientation of Beijing districts.

In 2017, Beijing issued The Beijing Urban Master Plan (2016–2030) and proposed to build a sustainable “green Beijing” in the new era. According to this plan, the districts of Beijing have a new functional orientation, including central urban districts and ecological conservation districts, as shown in Figure 2. Seven districts (Mentougou District, Pinggu District, Huairou District, Miyun District, Yanqing District, Changping District and Fangshan District) were selected as ecological conservation districts with stricter regulations on environmental protection and more support on ecological development. Compared with the central urban districts where medical resources are abundant and medical institutions are clustered, these districts suffer from a significant relative lack of medical resources due to their remote location. To provide accurate assessment and efficient improvement of primary healthcare resources, this study focuses on these 7 ecological conservation districts.

### 2.2. Data

**Figure 3:**
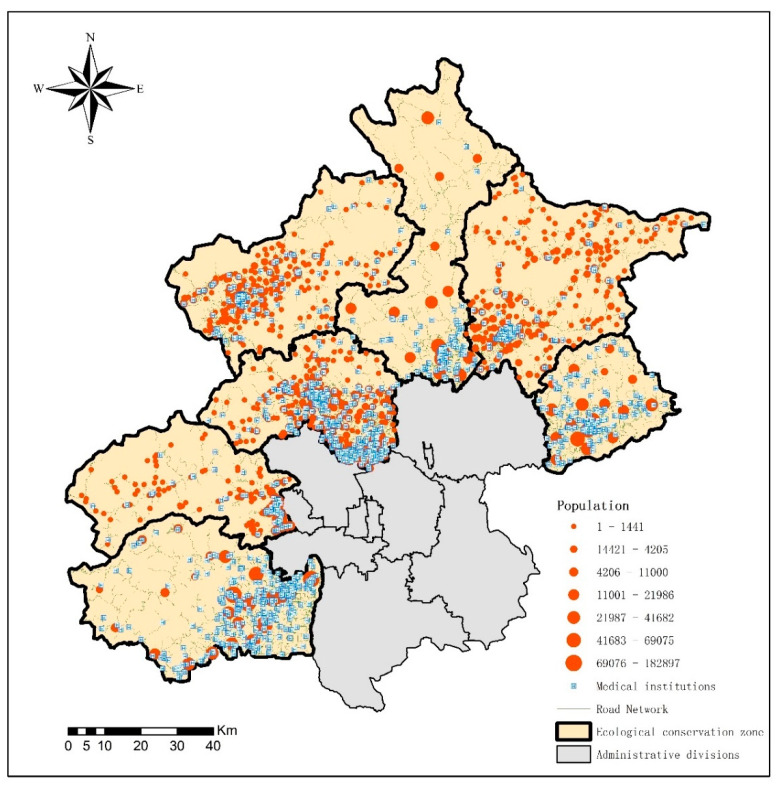
Distribution of primary health care institutions and population in the seven ecological conservation districts.

Data on primary health care institutions in 2016 were obtained from the Beijing Municipal Commission of Health and Family Planning, including the names, addresses, and the number of health professionals of the hospitals. The health professional includes chartered doctors, assistant chartered doctors, certified nurses, pharmacists, laboratory technicians and other professional medical workers, excluding hospital administrators. The road network and administrative boundary data in 2016 came from China’s National Basic Geographic Information System. Population data at the community level were obtained from the public security system in most districts except for Pinggu and Huairou Districts which were at the street level. Figure 3 shows the distribution of primary health care institutions and population in the seven ecological conservation districts.

### 2.3. Data Pretreatment

A GIS database built with ArcGIS 10.5 was established using the collected data mentioned above. Primary health care institutions mainly provide services such as diagnosis and treatment of common and frequently-occurring diseases, community-based rehabilitation care, and basic public health service. We used the number of health professionals because the focus of the services is not only on the diagnosis and treatment services and the primary health care institution services are mainly carried out by all health professionals rather than only the chartered doctors. The medical reception ability of the primary health care institutions was indicated as the number of health professionals. We calculated the travel time based on “The China Highway Technical Standards” which stipulates that each section of highway has a standard speed. Speed on expressways is specified as 120 km/h, 100 km/h for national highways, 80 km/h for provincial highways, 50 km/h for county roads, and 40 km/h for village roads. The walking speed is set as 5 km/h, and residents are required to walk to the nearest road before taking a car [14].

### 2.4. Lorenz Curve

The Lorenz curve is a curve reflecting the equity of social income distribution or property distribution, and it is now also widely used in research on the equity of health resource allocation [18,19]. The specific method is to use the cumulative percentage of population or area as the horizontal axis and the cumulative ratio of health resources as the ordinate and to draw the Lorentz curve by connecting the points. In the Lorentz curve graph, the diagonal line is the absolute fairness line. The closer the Lorentzian curve is to the complete fairness line, the better the fairness is; when the Lorentzian curve overlaps with the complete fairness line, the state of absolute fairness is reached.

### 2.5. Gini Coefficient

The Gini coefficient is a quantitative indicator based on the Lorenz curve examining the distribution of health resources against population size [20]. It measures deviations from a uniform distribution and indicates whether Beijing’s primary health resources are equally distributed across all communities [21]. The Gini coefficient of health resource allocation is calculated according to the following formula:

$$G=\sum_{i=1}^{n}X_{i}Y_{i}+2\sum_{i=1}^{n}X(1-V_{i})-1$$

In the formula, *X_i_* is the proportion of the population in each region to the total population; *Y_i_* is the proportion of a certain health resource index in each region to the total number of corresponding health resource indices; and *V_i_* = *Y*_1_ + *Y*_2_ + *Y*_3_ … *Y_i_* is the cumulative percentage of health resources. The Gini coefficient is between 0 and 1. A Gini coefficient of 0 indicates perfect fairness, while a Gini coefficient of 1 indicates extreme inequality [20]. At present, there is no specific evaluation standard for the Gini coefficient in the field of health, so they are all based on economic standards: a Gini coefficient below 0.2 is the best intermediate state, 0.3 to 0.4 is normal status, more than 0.4 is an alert status, and more than 0.6 is a highly unfair, dangerous status, which means that there is variability between populations. Spatial accessibility values of every community within a district were used to calculate the Gini Coefficient under the Rstudio 3.6 environment.

### 2.6. The Shortest Travel Time

The closest facility command under network analysis tools in ArcGIS 10.5 extension was used to calculate the closest primary health care institutions to each community. The speed of different roads is the same as the setting mentioned above. Because the policy requires 15 min to reach the nearest primary health care provider, we set the threshold at 15 min. Therefore, the shortest travel time was classified into three categories, 0–15, 15–30, and >30 min.

### 2.7. The Modified Two-Step Floating Catchment Area (M2SFCA) Method

Various algorithms have been promoted in available studies to measure the spatial accessibility of public services, gradually extending to assess the spatial accessibility of health services. Methods range from simple methods such as the supply-to-demand ratio and distance to the nearest facility to more complex methods such as the gravity model and two-step floating catchment area (2SFCA) method [22,23,24]. The 2SFCA method is essentially a special form of the gravity model, which is simpler to calculate and more intuitive to interpret [4,25]. This method has been used in several studies measuring the spatial accessibility of medical services [17,25,26]. However, the 2SFCA method has drawn increasing criticism because it defines a doctor inside a catchment as accessible and one outside the catchment as inaccessible [13]. Researchers have proposed an optimized method that added a Gaussian function to improve this model to overcome this limitation. The advantage of M2SFCA is that multiple distance attenuation weights replace dichotomies 0 and 1 in 2SFCA, which solves the problem of indistinguishable accessibility in the catchment [25,26,27].

Based on the distribution of the population and primary health care institutions, M2SFCA was used to further analyze the spatial accessibility of primary health care in Beijing. First, according to the relevant policy requirements of the state and the Beijing Municipality, the impedance parameter of the M2SFCA was selected. Therefore, we set a 15-min travel time as the impedance parameter of the M2SFCA in GIS when evaluating the spatial accessibility of primary health care services provided by primary health care institutions. The formula for determining accessibility is as follows:

$$A_{i}=\sum_{j=1}^{n}\frac{S_{j}f(d_{ij})}{\sum_{k=1}^{m}D_{k}f\left(d_{kj}\right)},f(d_{ij})={\{}_{0,d_{ij}>d_{0}}^{d_{ij}^{-\beta },d_{ij}\leq d_{0}}$$

*A_i_* indicated the spatial accessibility of population point *i* to a primary health care institutions, which in this study was calculated based on the data and the formula equal to the number of health professionals per 1000 population; *S_j_* was the capacity of the primary health care institutions to serve patients and was reflected in the number of health professionals; *D_k_* was the number of people in population point *k*; *d_ij_* was the travel time from population point *i* to health facility *j*; similarly, *d_kj_* was the travel time from population point *k* to health facility *j*; *d*_0_ was the threshold of the length of time used, which was 15-min in this study; n and m were the number of primary health care institutions and the number of population points, respectively; and *β* was the travel friction factor, usually between 1 and 2, taking into account the urgency of health services, resulting in a greater decay with increasing spatial distance [15,28]. Thus, in this research, *β* was 2.

## 3. Results

### 3.1. Gini Coefficient and Lorenz Curves

**Figure 4:**
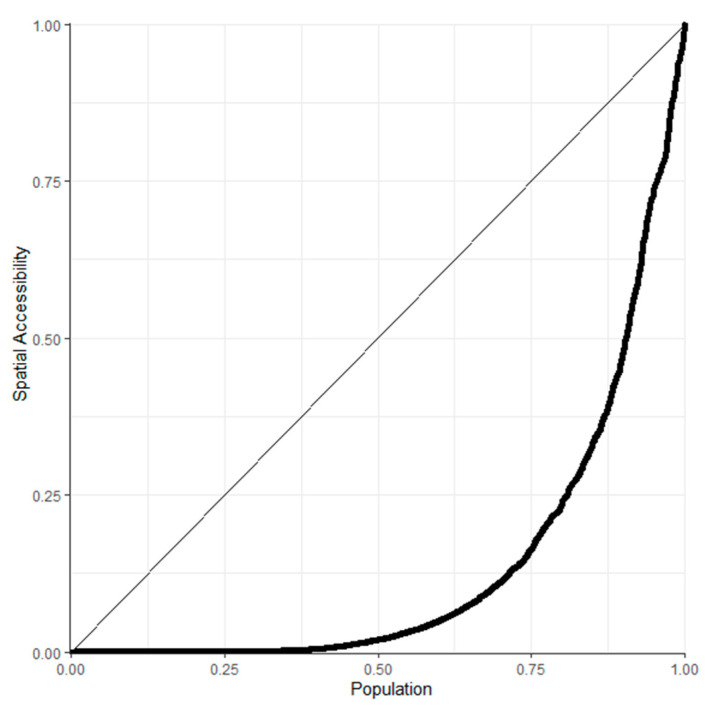
Lorenz curves for primary health care services by population in seven ecological conservation districts.

Figure 4 depicts the Lorenz curves for primary health care services by population. According to the figure, more than 75% of people shared only approximately 25% of the spatial accessibility, while the remaining less than 25% had three times as many resources as the former, indicating that the spatial distribution of medical resources in the area we studied is uneven. The Gini coefficient of primary medical services in seven ecological conservation districts was calculated to be as high as 0.705. The standards exceed the value of 0.4 mentioned above, which means a high level of unfairness. Therefore, it is necessary to redistribute medical resources in these areas based on a fairness model that enables the government to provide more equitable access to primary health care services.

### 3.2. Shortest Travel Time of Primary Health Care in Seven Ecological Conservation Districts

Table 1 presents a classification of the shortest travel time to primary health care facilities in the seven ecological conservation districts, including the percentage of communities and population. Similar results were obtained by calculating both communities and populations. Overall, 81.22% of communities reached the nearest primary care institution within 15 min, 11.55% did it in 15–30 min, and only 7.23% accomplished it in more than 30 min. Of the 124 communities that took more than 30 min, 58 were from Mentougou District, 40 were from Huairou District, and 15 were from Changping District. A total of 82.88% of the population arrived at the nearest primary health care institution within 15 min, 11.39% of the population reached it in 15–30 min, and 5.74% of the population reached it in more than 30 min. Of the population that arrived in more than 30 min, 37.4% were from Fangshan District, 33.0% were from Miyun District, and 18.2% were from Mentougou District.

**Figure 5:**
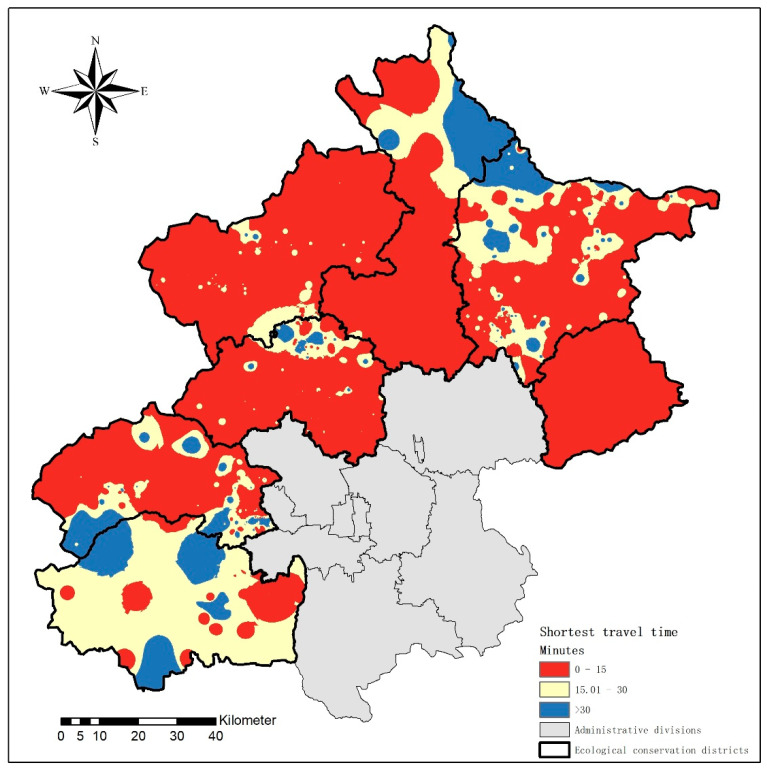
Shortest travel time to primary health care services by population in seven ecological conservation districts.

When focusing only on the relative geographic location of medical institutions and population points, as shown in Figure 5, most of the regions met the policy requirements. However, 10% of the communities were far from the nearest primary care institution, especially in Fangshan District, Mentougou District, and Miyun District.

### 3.3. Spatial Accessibility of Primary Health Care in Seven Ecological Conservation Districts

To indicate the accessibility of these areas quantitatively, we use common medical evaluation indicators. In 2016, the standard of the number of health professionals per 1000 people in Beijing was 12.19, and the average level of spatial accessibility of primary health care was 2.34 based on the ratio of the number of health professionals per 1000 people in primary health care institutions.

**Figure 6:**
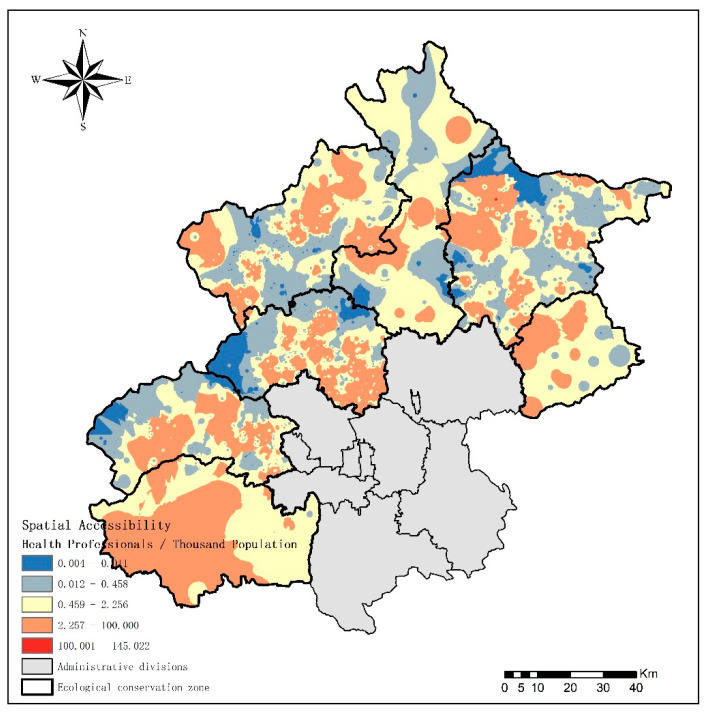
The spatial accessibility of primary health care services in seven ecological conservation districts.

In terms of access to health professionals (health professionals per 1000 people) in the 1715 communities, 183 (10.67% of the total) had scores higher than 9.92, and 391 (22.80%) had scores lower than 0.01. Table 2 displays a notable disparity in spatial accessibility within and between the seven ecological conservation districts. In the last 10% of communities and streets ranked as having the lowest primary health care spatial accessibility of the ecological conservation districts, Miyun, Mentougou, and Changping Districts were found to be the areas with the worst accessibility. The spatial accessibility of the seven districts is shown in Figure 6. The surrounding area of the district center has a prominently higher accessibility than that in other areas. The majority of the communities had relatively low accessibility of primary health care services, except a few communities where the primary health care institutions were located.

The results revealed that although Beijing is a city with relatively rich medical resources in China, there is still a large imbalance in the distribution of resources in some areas, and the gap between the surrounding areas of the city and the central city is relatively large. The cost of medical treatment for residents is relatively high, which means that there is still a gap between reality and the goals set by policymakers. At the government level, although each region calculates the corresponding target indicators when formulating the plan, it is not easy to continue to refine the indicators. The calculation results can provide specific evidence at the microsimulation level as the basis for allocating resources.

## 4. Discussion

This study examined the community-level accessibility of primary health care services in Beijing. Medical resource allocation planning takes into account a variety of factors, including equity and access to health care services. Spatial accessibility is a term that is increasingly being used in studies of health resource distribution, referring to citizens’ prospective capacity to obtain medical care. When calculating the time required for each village to reach the nearest primary health care institution, the shortest travel time was frequently selected. However, it considers just the spatial distance between the medical institution and the citizen region and ignores other factors that could affect demand for medical services, such as the health resources available at the medical institution. In comparison, the M2SFCA technique can consider both supply and demand factors, including the capacity of medical institutions, the population, and the state of transportation. Additionally, each community can calculate an accessibility score, which aids in the formulation of health policies and the improvement of previous planning completed using traditional methods. Finally, we calculated the Gini coefficient, a measure of inequities in various areas, by summing the accessibility scores by population. Although the results reflected only the broad characteristics of primary health care resources in ecological conservation districts, they provided an intuitive glimpse into resource allocations that were out of balance.

According to the results of the M2SFCA in seven ecological conservation districts, the spatial accessibility of primary health care services was at a low level, with nearly 65% of communities having a lower health workforce than the average level in Beijing. Among them, Miyun, Mentougou, and Changping Districts showed the lowest accessibility. According to several studies, deprived areas do not have a lower spatial accessibility to primary care [29]. In the majority of Australia’s non-metropolitan areas, which have exceptionally low population densities, health care services are inaccessible. The researchers discovered that remote locations received the most policy support, indicating that there were no apparent regional differences [30]. Our findings, however, contradict the findings of the aforementioned investigations. As was the case with previous evaluations of the spatial accessibility of health services undertaken in other provinces or cities, the deprived areas did have a lower level of accessibility. According to these analyses, the accessibility of primary health care services is at a low level nationwide and has significant regional disparities [17,31,32,33]. Most studies show that urban areas are better than rural areas and plain areas are better than mountainous areas, which could be the result of differences in economy, geographical environment, and social development. The region we chose is the economically underdeveloped areas of Beijing, and the results also revealed poorer accessibility. Additionally, this indicates a general lack of knowledge of a city’s surrounding territory, which has a lower development value and a smaller population. These locations, however, are crucial for urban construction and resource decongestion. By comparing the spatial accessibility of hospitals in Michigan, M2SFCA is more effective than several other methods of calculating accessibility and can avoid overestimation [34].

Although Beijing’s primary care institutions are generally well established, primary health care capacity remains an issue, particularly in ecological conservation districts. The comparatively low economic and social development level, combined with the mountainous natural geographical setting, may impede or even obstruct the successful establishment and growth of a health service system, resulting in a relatively low degree of primary medical care accessibility. To a certain extent, this study can guide policymakers on how to conduct more innovative health service planning. To begin, by assessing the Gini coefficient and computing spatial accessibility, policymakers can identify inequities in primary health care services and allocate resources more precisely to less accessible and underserved places. Second, present planning methods emphasize measuring health care resource availability within administrative boundaries, although patient consumption of primary health care services is mostly affected by distance. Accessibility determined by the use of particular distance threshold will enable policymakers to place a greater emphasis on actual patient demand, which was not considered in the Health Planning. Additionally, the results of the accessibility study can help policymakers augment and improve previous planning methodologies that focused exclusively on medical resources divided by population. To improve primary health care accessibility in Beijing, the government can close the gap between districts by improving road conditions, increasing investment in primary health care, increasing the number of primary health care professionals, and promoting a more balanced distribution of medical resources in order to achieve the goal of universal primary health care services and guarantee each citizen can get proper and accessible treatment. Numerous regional health plans advocate expanding primary care facilities in heavily populated areas, and this technique could be utilized to increase accessibility in Beijing as well.

To our knowledge, this is the first study to assess primary care service accessibility in Beijing using community-level populations. Previous studies of China’s medical accessibility that used the M2SFCA technique did not include Beijing, a region with relatively ample medical resources but a huge disparity in medical resource. Beijing’s distribution of health resources will serve as a model for other regions, as it is a pioneer city in China’s medical planning. Additionally, as the capital city, Beijing’s actions have garnered greater attention from the Chinese central government in terms of urban resource relief. The M2SFCA approach is used to evaluate this region because it is one of the current mainstream evaluation methods, and the outcome is quite dependable. The evaluation’s findings demonstrate that present accessibility in and around Beijing is considerably diverse and that additional efforts are needed to ensure the equitable distribution of medical services. Fortunately, the results established using the M2SFCA approach have been implemented in some areas, according to planning documents provided by the People’s Government of Beijing Municipality. This result is not mentioned in the previous research, and it is also a manifestation of the practical application of our research results. Additionally, study on the accessibility of primary health care resources in the capital may yield similar insights for other developing countries. Ample resources do not guarantee that everyone gets access to medical care that is appropriate for their circumstances. Additional evidence supporting the rationality of primary health care allocations is required, and the M2SFCA technique is an excellent alternative. Combining spatial accessibility with health is an exciting area that has the potential to fill resource allocation gaps.

Several limitations apply to this research. First, this is a cross-sectional study since data on the community population were only available in 2016; consequently, data on primary care institutions were limited in 2016 for data matching, which may impair the reliability of the results and diminish their explanatory power. However, Beijing’s primary health care institutions have changed very little from 2016 to 2021, increasing from 9676 to 10,183, mainly in urban areas. Therefore, the results may provide advice for policymaking, and the results have already been confirmed by the medical resource allocation policies issued by the health administration later, playing the expected role. Correspondingly, following the acquisition of additional data, other research might do a comparative analysis of time trends. Second, in this study, only the Lorenz curve was used to assess inequity. More indicators based on spatial econometrics could be incorporated in subsequent studies to quantitatively bolster the findings. 

## 5. Conclusions

Over 80% of the population meets Beijing’s 15-min health resource allocation goal. This means that people can get medical attention immediately if they are sick, which is a basic guarantee for residents’ health. However, only describing the adequacy and accessibility of medical supplies in terms of distance is insufficient. The results from the M2SFCA analysis revealed more detailed spatial accessibility disparities in the 1715 communities, which should be given great attention. To summarize, the findings above can aid in the planning of healthcare service resource allocation in the study area and guide officers to place a greater emphasis on arrangements other than numbers. These areas demand enhanced standardization of primary medical and health institutions, increased emphasis on diagnosing common and frequently occurring diseases, and increased capacity for key medical and health services provided by community health service centers and village clinics.

## Figures and Tables

**Table 1:** Shortest travel time in seven ecological conservation districts.

Shortest Travel Time (Minutes)		All	Changping	Fangshan	Huairou	Mentougou	Miyun	Pinggu	Yanqing
0–15	Communities (%)	81.22	90.52	56.00	88.24	77.78	66.58	100	86.17
	Population (%)	82.88	92.48	71.66	96.24	79.79	72.88	100	86.77
15–30	Communities (%)	11.55	6.69	28.00	0	9.15	18.84	0	12.62
	Population (%)	11.39	6.22	21.45	0	8.27	13.77	0	12.64
>30	Communities (%)	7.23	2.79	16.00	11.76	13.07	14.58	0	1.21
	Population (%)	5.74	1.30	6.89	3.76	11.94	13.35	0	0.58

**Table 2:** Spatial accessibility of seven ecological conservation districts.

Spatial Accessibility	Max	Min	Median	Q1 ^a^	Q3 ^a^
Changping	84.91	<0.01	1.93	0.16	5.09
Fangshan	6.35	0.18	2.27	1.34	3.33
Huairou	5.05	<0.01	1.97	<0.01	2.61
Mentougou	69.36	<0.01	0.69	0.01	2.29
Miyun	46.95	<0.01	0.27	<0.01	1.74
Pinggu	6.48	<0.01	1.33	<0.01	3.29
Yanqing	76.25	<0.01	0.47	0.02	2.86

^a^ We ranked the results for spatial accessibility in descending order. Q1 means the first quartile. Q3 means the third quartile.

## Data Availability

The datasets used or analysed during the current study are available from the corresponding author on reasonable request.
